# The Early Predictive Value of Circulating Monocytes and Eosinophils in Coronary DES Restenosis

**DOI:** 10.3389/fcvm.2022.764622

**Published:** 2022-02-22

**Authors:** Shumei Li, Hong Qiu, Zhaorong Lin, Lin Fan, Yongzhe Guo, Yujie Zhang, Lianglong Chen

**Affiliations:** ^1^Department of Cardiology, Fujian Institute of Coronary Heart Disease, Fujian Medical University Union Hospital, Fuzhou, China; ^2^JC School of Public Health and Primary Care, The Chinese University of Hong Kong, Shatin, Hong Kong SAR, China

**Keywords:** monocyte, eosinophil, in-stent restenosis, drug-eluting stent, coronary heart disease

## Abstract

**Background:**

Monocytes and eosinophils are involved in intracoronary inflammatory responses, aggravating coronary artery plaque instability and in-stent restenosis (ISR).

**Aims:**

To investigate an early prediction of ISR in patients undergoing stenting by circulating monocytes and eosinophils.

**Methods:**

The single-center data of patients undergoing successful drug-eluting stents (DES) implantation from January 1, 2017 to April 30, 2020 were retrospectively analyzed. Of the 4,392 patients assessed, 140 patients with restenosis and 141 patients without restenosis were enrolled. A scheduled postoperative follow-up was proceeded in four sessions: 0–3 months, 3–6 months, 6–12 months, and >12 months. The hematological and biochemical measurement was collected. The angiographic review was completed within two postoperative years.

**Results:**

Significant associations of monocyte count and percentage with ISR were evident [odds ratio (OR): 1.44, 95% CI: 1.23–1.68, *P* < 0.001; OR: 1.47, 95%CI: 1.24–1.74, *P* < 0.001, respectively], which began at 3 months postoperatively and persisted throughout the follow-up period. Eosinophil count and percentage were associated with ISR (OR: 1.22, 95%CI: 1.09–1.36, *P* = 0.001; OR: 1.23, 95%CI: 1.07–1.40, *P* = 0.003, respectively), with ISR most significantly associated with the baseline eosinophils. The receiver operating characteristic (ROC) curve analysis showed that the cutoff points of monocyte count and percentage in the ISR prediction were 0.46× 10^9^/L and 7.4%, respectively, and those of eosinophil count and percentage were 0.20 × 10^9^/L and 2.5%, respectively.

**Conclusion:**

This study, with a long-term follow-up, first provides evidence that the elevated monocytes at three postoperative months and baseline eosinophils may be strong early predictors of ISR after drug-eluting stent implantation. Persistent elevation of monocytes may also be a signal of ISR after percutaneous coronary intervention (PCI).

## Introduction

Although percutaneous coronary intervention (PCI), a widely-prescribed treatment for symptomatic coronary disease, has proven effective, in-stent restenosis (ISR) remains a clinical challenge. It may result in repeated revascularization and poor long-term prognosis for afflicted patients, despite the reduced neointimal hyperplasia and incidence rate of <10% due to the emergence of drug-eluting stents (DES) (1, 2).

As a key feature of atherosclerosis, inflammation represents a well-known pathogenic mechanism underlying both coronary plaque progression and instability and adverse events following stent implantation. Available evidence suggests that effector cells of allergic inflammation such as eosinophils, as well as classic inflammatory cells, including monocytes, macrophages, lymphocytes, and neutrophils, play an important role in ISR (3–5).

Several studies have investigated whether laboratory parameters of complete blood count or biochemical analysis could predict ISR, but yielded inconsistent results (6–12). Moreover, previous studies only employed bare-metal stent (BMS) and blood samples from the peri-interventional period and did not supplement with a dynamic follow-up. In the current study, we monitored the serial changes of monocytes and eosinophils by a simple blood draw after stent implantation, attempting to investigate whether circulating monocytes and eosinophils can identify the ISR in those patients undergoing stenting at an early phase.

## Methods

### Study Population

Patients, who had been diagnosed with stable angina pectoris or acute coronary syndrome and underwent successful DES implantation during January 1, 2017 and April 30, 2020, were enrolled at the Affiliated Union Hospital of Fujian Medical University. The excluding criteria were as follows: no follow-up coronary angiography (CAG) within 2 years after PCI, requiring PCI for other vessels at the follow-up visit, severe hepatic and renal diseases, thyroid disease, malignancy, allergic diseases, hematological disorders, immunological disorder, and active infection. ISR referred to the plaque within 5 mm of the edge of the stent after PCI and had stenosis >50% (13). Of the 4,392 patients who underwent PCI during this study period, 140 patients with restenosis met the inclusion criteria, and 141 patients without restenosis in the same period were selected randomly as controls. Patients were advised to return for scheduled postoperative follow-up analysis during 4 periods: 0–3 months, 3–6 months, 6–12 months, and >12 months.

The study protocol observed the recommendations of the Declaration of Helsinki on Biomedical Research involving human subjects and was approved by the institutional ethics committee of Fujian medical university Union Hospital (Ethics approval number: 2021KY080). The written informed consent was obtained from all study participants.

### Measurements of Blood Parameters

Blood samples were collected during the clinical visit. Specimens were used for hematological and biochemical measurement. Total white blood cells and each fraction were measured with an automated hematology analyzer (Sysmex XN2000, Japan). Plasma total cholesterol, low-density lipoprotein cholesterol, high-density lipoprotein cholesterol, triglyceride, apolipoprotein A, apolipoprotein B, creatinine, and uric acid were analyzed in fasting blood samples with an automatic biochemical detector (Cobas 8000, Roche, Germany). High-sensitivity C-reactive protein was determined by immune turbidimetry with an analyzer (Beckman immage800, USA). HbA1c, as a marker of glycemic control level, was detected on a glycosylated hemoglobin analyzer (Sysmex G7, Japan). Serum homocysteine (HCY) was quantified by the chemiluminescent method with Architect i2000sr (Abbott, USA).

### In-stent Restenosis Assessment

For all patients, the PCI procedure and the implantation of DES were performed according to the PCI guideline. For patients without clinical contraindications, coronary angiography was routinely performed within the 2-year follow-up. Angiograms were analyzed with a validated quantitative coronary angiography system (Philips UNIQ FD20, Holland). Angiographic restenosis was defined as percent diameter stenosis >50% during the follow-up and the patients were accordingly divided into the ISR and non-ISR groups.

### Statistical Analyses

The essential characteristics and the hematological and biochemical indices were compared between postoperative in-stent restenosis (ISR) and non-ISR group. The continuous variables with approximately normal distribution were presented as mean and SD, and compared by independent samples *t*-test; continuous variables with skewed distribution were described as median (25th, 75th percentiles) and compared by Mann-Whitney *U*-Test; categorical variables were described as the frequency with percentage and compared by the Chi-square test.

As it is a case-control study design with longitudinal repeated measurements of hematological and biochemical indices, we applied mixed-effect logistic regression in the generalized linear mixed model family to examine the associations of each hematological and biochemical parameter with the risk of ISR. The follow-up month (times of repeated measurements) was included as a random intercept in the model to account for the within-subject correlation, and individual essential characteristics at baseline (age, sex, BMI, smoking status, chronic diseases of hypertension, or diabetes mellitus) as fixed effects to adjust the between-subject confounders (14, 15).

In order to evaluate whether the main hematological indices (monocyte and eosinophil count/percentage) have an early prediction of ISR or not, we conducted a follow-up period-specific analysis. The logistic regression in the generalized linear model family was applied to examine the risk of ISR associated with each of the main hematological indices during each follow-up period, with the individual essential characteristics adjusted.

The optimal cutoff points for monocyte and eosinophil count/percentage in the prediction of ISR were identified according to the receiver operating characteristic (ROC) curve analysis (16). The optimal cutoff points were then used to categorize monocyte and eosinophil count/percentage into low and high levels, respectively, and examine their joint effect and potential biological interaction (17). The predictive values of monocyte and eosinophil count/percentage were also evaluated by the area under the ROC curve (AUC) (18).

All analyses were conducted within the R statistical environment version 3.5.3 using the “lme4” package for generalized mixed effect model, and “cutpointr” and “ModelGood” packages to identify the optimal cutoff point and to obtain ROC curves based on the logistic regression (19).

## Results

The comparison of clinical characteristics between the ISR and non-ISR groups was summarized in Table 1.

**Table 1:** Data description of basic characteristics and main hematological and biochemical indices between postoperative in-stent restenosis (ISR) and non-ISR group^*^.

	**ISR group**	**Non-ISR group**	***P*-value**
**Basic characteristics**	***N*** **=** **140**	***N*** **=** **141**	
Age	65.1 ± 10.1	61.8 ± 9.9	<0.001
Sex, men	115 (82.1%)	106 (75.2)	0.201
BMI	23.9 ± 3.0□	24.4 ± 2.9	<0.001
Smoking			0.857
Never-smoker	73 (52.1%)	71 (50.4%)	
Ever-smoker	67 (47.9%)	70 (49.6%)	
Hypertension	97 (69.3%)	86 (61.0%)	0.182
Diabetes Mellitus	70 (50.0%)	41 (29.1%)	<0.001
**Hematological and biochemical** **indices with repeated measurements**	*n* = 507	n = 594	
White blood cell count (× 10^9^/L)	7.054 ± 2.066	6.942 ± 1.903	0.348
Neutrophil count (× 10^9^/L)	4.452 ± 1.713	4.486 ± 2.932	0.818
Lymphocyte count (× 10^9^/L)	1.859 ± 0.654	1.893 ± 0.661	0.386
Monocyte count (× 10^9^/L)	0.525 ± 0.202	0.469 ± 0.181	<0.001
Eosinophil count (× 10^9^/L)	0.189 ± 0.182	0.151 ± 0.140	<0.001
Neutrophil percentage (%)	63.41 ± 30.88	61.95 ± 8.69	0.303
Lymphocyte percentage (%)	26.99 ± 7.80	28.31 ± 8.46	0.007
Monocyte percentage (%)	7.53 ± 2.07	6.83 ± 1.87	<0.001
Eosinophil percentage (%)	2.70 ± 2.25	2.24 ± 1.74	<0.001
Total cholesterol (mmol/L)	3.732 ± 1.022	3.913 ± 1.097	0.005
Low-density lipoprotein cholesterol (mmol/L)	2.216 ± 0.882	2.374 ± 0.970	0.005
High-density lipoprotein cholesterol (mmol/L)	1.024 ± 0.264	1.082 ± 0.297	<0.001
Triglyceride (mmol/L)	1.791 ± 1.380	1.734 ± 1.030	0.450
Apolipoprotein A (g/L)	1.202 ± 0.268	1.235 ± 0.241	0.030
Apolipoprotein B (g/L)	0.816 ± 0.258	0.841 ± 0.268	0.122
Serum creatinine (μmol/L)	83.38 ± 21.09	81.94 ± 21.21	0.268
Estimated glomerular filtration rate (mL/min/1.73m^2^)	71.46 ± 26.16	79.54 ± 21.60	<0.001
Serum uric acid (μmol/L)	384.75 ± 101.18	375.97 ± 111.27	0.177
High-sensitivity C-reactive protein (mg/L)^#^	1.71 (0.51, 5.90)	1.55 (0.45, 5.35)	0.441
HBA1c (%)	7.22 ± 1.75	6.63 ± 1.33	<0.001
Homocysteine (umol/L)	10.51 ± 4.61	10.05 ± 5.50	0.320

* *Continuous variables with approximately normal distribution are described as Mean±SD, and compared with independent samples t-test; Categorical variables were described as N (%) and compared with Chi-square test*.

# *Continuous variables with skewed distribution were described as median (25th, 75th percentiles), and compared with Mann-Whitney U-Test*.

**Figure 1:**
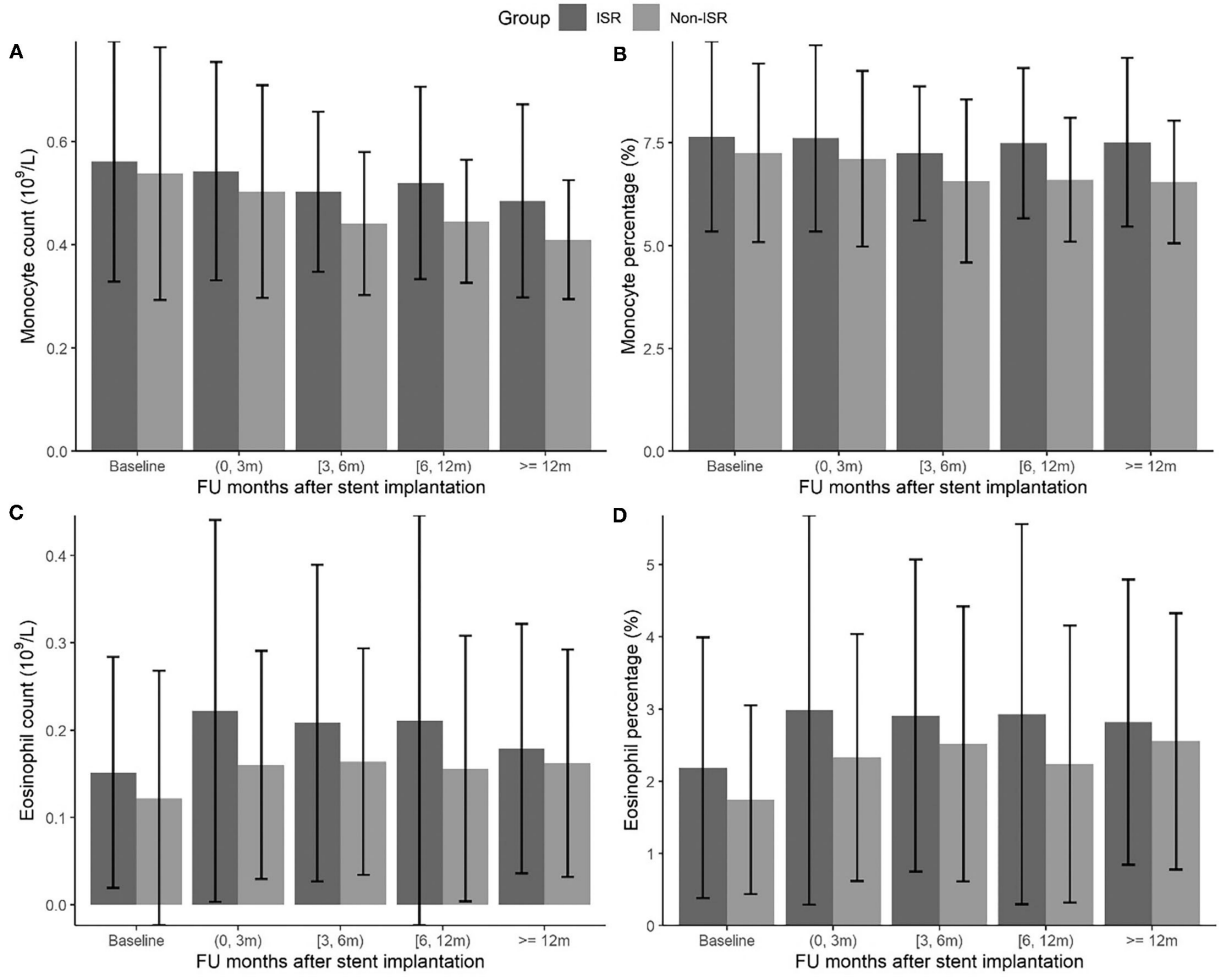
The changes of monocyte and eosinophil count (10^9^/L) **(A,C)** and percentage (%) **(B,D)** during the follow-up months between ISR and non-ISR groups.

The effect estimates were presented as the odds ratio (OR) of ISR with the 95% CI associated with an interquartile range (IQR) increment of each hematological and biochemical index. We observed the statistically significant associations of ISR with monocyte and eosinophil count and percentage, respectively (Table 2). The OR of ISR was 1.44 (95%CI: 1.23–1.68, *P* < 0.001) per IQR increase of monocyte count (0.21 × 10^9^/L) and 1.47 (95%CI: 1.24–1.74, *P* < 0.001) per IQR increase of monocyte percentage (2.42%). The follow-up period-specific analysis (Table 3) showed that significant associations began at 3 months after the operative intervention and persisted throughout the following observation period, which suggests an early prediction of ISR by monocytes. Meanwhile, the OR of ISR was 1.22 (95%CI: 1.09–1.36, *P* = 0.001) per IQR increase of eosinophil count (0.13 × 10^9^/L) and 1.23 (95%CI: 1.07–1.40, *P* = 0.003) per IQR increase of eosinophil percentage (2.00%). The most significant association between ISR and eosinophils occurred at baseline and lasted for about 1 year, supporting the early prediction of ISR by eosinophils. The mean levels of monocytes and eosinophil percentage and count at each follow-up period between ISR and non-ISR groups supported the above-mentioned observations (Figure 1). Besides, lower high-density lipoprotein cholesterol (OR = 0.8, 95%CI.67–0.95, *P* = 0.012) and higher HbA1c (OR = 1.28, 95%CI 1.03–1.60, *P*= 0.025) raised the ISR risk. Other parameters did not show a significant correlation with ISR (Table 2).

**Table 2:** OR (95%CI) of ISR associated with per interquartile range (IQR) increment of each hematological and biochemical index^*^.

**Hematological and biochemical indices**	**IQR**	**OR (95% CI)**	***P*-value**
White blood cell count (× 10^9^/L)	2.52	1.18 (1.00, 1.40)	0.052
Neutrophil count (× 10^9^/L)	1.91	1.01 (0.91, 1.11)	0.916
Lymphocyte count (× 10^9^/L)	0.77	1.15 (0.99, 1.35)	0.074
Monocyte count (× 10^9^/L)	0.21	**1.44 (1.23, 1.68)**	<0.001
Eosinophil count (× 10^9/^L)	0.13	**1.22 (1.09, 1.36)**	0.001
Neutrophil percentage (%)	11.49	1.04 (0.98, 1.12)	0.209
Lymphocyte percentage (%)	10.96	0.94 (0.79, 1.13)	0.525
Monocyte percentage (%)	2.42	**1.47 (1.24, 1.74)**	<0.001
Eosinophil percentage (%)	2.00	**1.23 (1.07, 1.40)**	0.003
Total cholesterol (mmol/L)	1.34	0.89 (0.75, 1.05)	0.169
Low-density lipoprotein cholesterol (mmol/L)	1.19	0.90 (0.76, 1.06)	0.211
High-density lipoprotein cholesterol (mmol/L)	0.34	**0.80 (0.67, 0.95)**	0.012
Triglyceride (mmol/L)	0.92	1.04 (0.94, 1.16)	0.417
Apolipoprotein A (g/L)	0.31	0.88 (0.75, 1.04)	0.137
Apolipoprotein B (g/L)	0.32	1.02 (0.87, 1.19)	0.832
Serum creatinine (μmol/L)	23.00	0.94 (0.80, 1.09)	0.408
Estimated glomerular filtration rate (mL/min/1.73 m^2^)	31.42	0.89 (0.71, 1.11)	0.309
Serum uric acid (μmol/L)	126.00	1.12 (0.96, 1.32)	0.142
High-sensitivity C-reactive protein (mg/L)	5.12	0.97 (0.91, 1.03)	0.260
HBA1c (%)	1.60	**1.28 (1.03, 1.60)**	0.025
Homocysteine (umol/L)	3.59	1.07 (0.93, 1.23)	0.344

* *Generalized linear mixed models (mixed-effects logistic regression) were used to examine the odds ratio (ORs) of ISR with the hematological and biochemical indices, adjusting for follow-up time as a random effect and individual basic characteristics (age, sex, BMI, smoking status, chronic diseases of hypertension or diabetes mellitus) as fixed effects. Each of the blood biochemical indices was included into the model one at a time. Statistically significant effect estimates are in bold*.

**Table 3:** OR (95%CI) of ISR associated with per interquartile range (IQR) increment of each hematological index during the follow-up period^*^.

	**ISR**	**Non-ISR**	**OR (95% CI)**	***P*-value**
Monocyte count (10^9^/L)^#^	507	594	**1.44 (1.23, 1.68)**	<0.001
Baseline	140	141	1.13 (0.89, 1.42)	0.313
>0 & <3 M	87	107	1.22 (0.87, 1.70)	0.244
≥3 & <6 M	69	72	**2.35 (1.25, 4.42)**	0.008
≥6 & <12 M	99	141	**2.25 (1.38, 3.68)**	0.001
≥12 M	112	133	**2.26 (1.44, 3.56)**	<0.001
Monocyte percentage (%)^#^	507	594	**1.47 (1.24, 1.74)**	<0.001
Baseline	140	141	1.16 (0.87, 1.55)	0.317
>0 & <3 M	87	107	1.25 (0.86, 1.81)	0.242
≥3 & <6 M	69	72	**1.82 (1.04, 3.17)**	0.034
≥6 & <12 M	99	141	**2.03 (1.23, 3.33)**	0.005
≥12 M	112	133	**2.02 (1.33, 3.08)**	0.001
Eosinophil count (10^9^/L)^#^	507	594	**1.22 (1.09, 1.36)**	0.001
Baseline	140	141	**1.30 (1.00, 1.70)**	0.050
>0 & <3 M	87	107	1.31 (0.99, 1.73)	0.057
≥3 & <6 M	69	72	1.30 (0.94, 1.81)	0.109
≥6 & <12 M	99	141	1.18 (0.92, 1.51)	0.185
≥12 M	112	133	1.14 (0.88, 1.47)	0.334
Eosinophil percentage (%)^#^	507	594	**1.23 (1.07, 1.40)**	0.003
Baseline	140	141	**1.49 (1.06, 2.10)**	0.021
>0 & <3 M	87	107	1.25 (0.92, 1.70)	0.155
≥3 & <6 M	69	72	1.24 (0.86, 1.78)	0.251
≥6 & <12 M	99	141	1.25 (0.92, 1.70)	0.159
≥12 M	112	133	1.11 (0.83, 1.49)	0.465

* *Generalized linear models (logistic regression) were used to examine the odds ratio (ORs) of ISR with an IQR increment of the hematological indices during each follow-up period, adjusting for individual basic characteristics including age, sex, BMI, smoking status, chronic diseases of hypertension and diabetes mellitus. The IQR for monocyte and eosinophil count was 0.21 × 10^9^/L and 0.13 × 10^9^/L, respectively; and the IQR for monocyte and eosinophil percentage was 2.42% and 2.00%, respectively*.

# *ORs were estimated from the generalized linear mixed models, as those in Table 2. Each of the blood biochemical indices was included into the model one at a time. Statistically significant effect estimates are in bold*.

**Figure 2:**
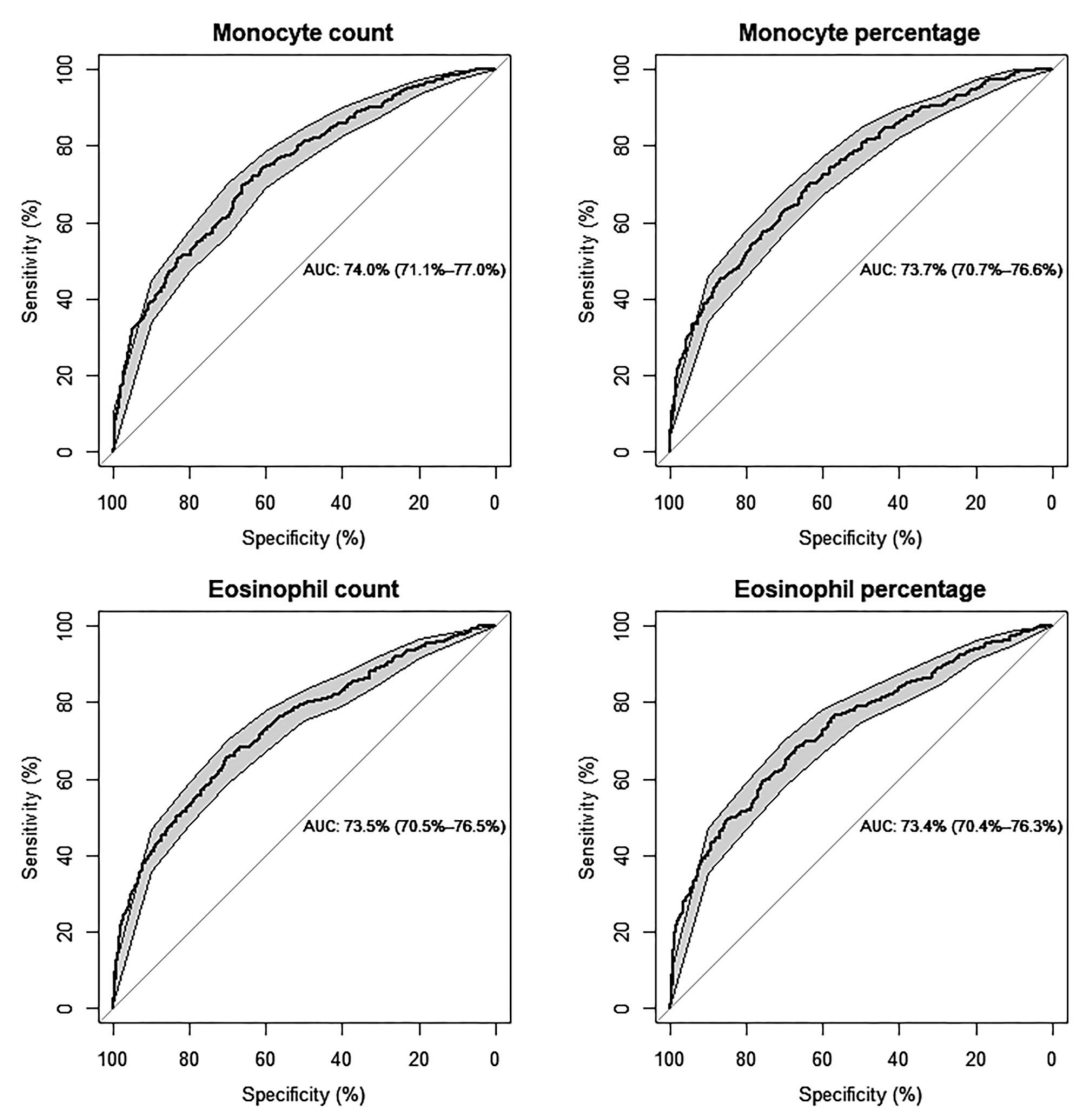
Predictive values of monocyte and eosinophil count (10^9^/L) and percentage (%) for the risk of ISR, respectively, by ROC curve analysis (Models are adjusted for individual basic characteristics, including age, sex, BMI, smoking status, chronic diseases of hypertension and diabetes mellitus, and follow-up time. AUC: area under the curve with 95% confidence interval).

The ROC and AUC were employed to explore the diagnostic value of monocyte and eosinophil count and percentage in ISR prediction. On ROC analysis, the optimal cutoff points were identified as 0.46 × 10^9^/L and 7.4% for monocyte count and percentage, respectively (AUC: 74%, 95% CI: 71.1–77%, *P* < 0.001; AUC: 73.7%, 95% CI: 70.7–76.6%, *P* < 0.001). The optimal cutoff points were identified as 0.20 × 10^9^/L and 2.5% for eosinophil count and percentage, respectively (AUC: 73.5%, 95% CI: 70.5–76.5%, *P* < 0.001; AUC: 73.4%, 95% CI: 70.4–76.3%, *P* < 0.001) (Figure 2).

Furthermore, we observed the joint effect of monocyte and eosinophil count and percentage on the risk of postoperative ISR. The value beyond the cutoff points was defined as high levels. When both monocyte and eosinophil count and percentage were high (OR of ISR: 3.04, 95%CI: 2.06–4.49, *P* < 0.001 for the joint count; OR of ISR: 3.06, 95%CI: 2.08–4.51, *P* < 0.001 for joint percentage), the risk of ISR was higher than that of a single index (Table 4).

**Table 4:** The joint effect of monocyte and eosinophil Count or percentage on the risk of postoperative in-stent restenosis (ISR).

**Joint effect**	**ISR (*n* = 507)**	**Non-ISR (*n* = 594)**	OR (95% CI)^*^	***P*-value**
Monocyte and eosinophil Count^a^				
Both monocyte and eosinophil count are low	153 (30.2)	292 (49.3)	1.00	-
Only monocyte count is high	180 (35.5)	167 (28.2)	2.21 (1.61, 3.04)	<0.001
Only eosinophil cunt is high	49 (9.7)	57 (9.6)	1.82 (1.14, 2.91)	0.012
Both monocyte and eosinophil count are high	125 (24.7)	76 (12.8)	3.04 (2.06, 4.49)	<0.001
Monocyte and eosinophil Percentage^b^				
Both monocyte% and eosinophil% are low	149 (29.4)	273 (46.1)	1.00	-
Only monocyte% is high	135 (26.6)	125 (21.1)	1.85 (1.30, 2.62)	<0.001
Only eosinophil% is high	95 (18.7)	125 (21.1)	1.32 (0.92, 1.91)	0.135
Both monocyte% and eosinophil% are high	128 (25.2)	69 (11.7)	3.06 (2.08, 4.51)	<0.001

* *Generalized linear mixed models (mixed-effects logistic regression) were used to examine the odds ratio (ORs) of ISR*.

a *The optimal cutoff points were identified as 0.46 × 10^9^/L and 0.20 × 10^9^/L for monocyte and eosinophil count, respectively, to categorize them into low and high levels*.

b *The optimal cutoff points were identified as 7.4% and 2.5% for monocyte and eosinophil percentage, respectively, to categorize them into low and high levels*.

## Discussion

Inflammation is an important player both for the initiation and progression of coronary artery disease and for coronary plaque instability. Moreover, experimental studies have demonstrated that local and systemic inflammation may promote neointimal proliferation, which serves as the leading mechanism involved in the pathogenesis of ISR.

Classic inflammatory cells such as monocytes have been demonstrated to infiltrate into and accumulate to the stenting site, secreting numerous growth factors, cytokines and promoting the migration and proliferation of vascular smooth muscle cells (SMCs) to the subendothelial space. Some studies believe that the activated monocytes may differentiate into the neointimal SMCs, becoming a component of the neointima (6, 20). Hong YJ and Fukuda D et al. reported that circulating pre-interventional monocyte count may be related to in-stent neointimal volume (6, 7). But no serial blood parameters have been monitored in these studies. Intimal hyperplasia after BMS usually peaks between 6 months−1 year, after which a quiescent period resumes (21, 22); however, this typically occurs earlier within 6 months of stenting in DES (23, 24). Different from previous studies, our results indicate that monocyte count and percentage within 3 months after stent implantation had no predictive value for restenosis. The increase of monocytes in both groups may be related to plaque rupture, inflammation activation, and endothelial repair after stenting. Significant associations of ISR with monocyte percentage and count began at 3 months and lasted for more than 1 year, which suggests the strong early prediction of ISR by monocytes. The optimal cutoff points of monocyte count and percentage were 0.46 (10^9^/L) and 7.4%, respectively.

Besides classic inflammation, mounting evidence derived from both experimental and clinical studies suggests an important, yet under-recognized, role for effector cells of allergic inflammation in both the pathogenesis of coronary artery disease and adverse events following stent implantation. Eosinophils may promote thrombus formation, endothelial damaging and coronary plaque instability (5). The metal stent struts and the polymer may trigger local recruitment and activation of allergic inflammation. Histopathologic studies showed that eosinophils were observed to infiltrate into DES at a higher concentration when compared with BMS (25, 26). These findings suggest that allergy-mediated inflammation plays a greater role in DES-related ISR.

Eosinophil count, in epidemiological studies, has been associated with future ischemic heart disease (IHD) (27). Eotaxin, a potent eosinophil chemokine, has been involved in an increased coronary atherosclerotic burden (28) and the baseline serum levels of ECP, a sensitive marker of eosinophil activation can predict the clinical outcome after the implantation of first-generation DES (29). Inconsistencies still arise with regards to the association between eosinophil count and ISR. Hajizadeh R et al. reported that blood eosinophil count measured 6 weeks after PCI has a significant association with the development of ISR within 6-month after DES implantation (30). However, Verdoia M et al. argued that eosinophils levels are not independently associated with the prevalence and extent of coronary artery disease (31).

Different from the above findings, we demonstrated a higher prevalence of ISR in patients with a blood eosinophil count >0.20 (10^9^/L) and a percentage >2.5% (*P* = 0.001, *P* = 0.003 respectively) at baseline. In the supplementary materials, we furthermore analyzed the changes of eosinophils in two groups before and after operation separately. The levels of postinterventional eosinophils in ISR and non-ISR groups were both higher than those at baseline, with the increase in the ISR group significantly greater than that in the non-ISR group (Supplementary Figure 1). This result supports the induction of allergic inflammation after stenting. For the insignificant association between ISR and postinterventional eosinophil count and percentage, a possible explanation may lie in the relatively small sample size. Accordingly, our results show that enhanced eosinophilic activation at baseline or post-intervention may possess an early predictive value for ISR.

We also confirmed that low HDL cholesterol increased ISR rates, which is consistent with the finding that HDL cholesterol enhances stent biocompatibility (32). Similar results have also been reported in patients with diabetes with coronary heart disease and carotid artery stents implantation (33, 34).

Diabetes mellitus (DM) has been consistently found to be an independent risk factor for poor outcomes following PCI in several previous studies (35). Although the introduction of DES reduces the restenosis rates, diabetic patients as a group continue to experience poor outcomes (36). Although with currently available therapies, some believe that DM is not a risk factor for poor outcomes following DES (37, 38), in our study, HbA1c is still an independent risk factor for ISR, which is in agreement with most previous studies.

In conclusion, our study demonstrates that circulating monocytes at 3 months after DES implantation and baseline eosinophils can strongly predict the risk of ISR. Persistent elevation of monocytes also may be a signal of ISR after PCI. Lower high-density lipoprotein cholesterol and increased HbA1c are significantly associated with ISR. Persistent elevation of monocytes also may be a signal of ISR after PCI.

## Data Availability Statement

The data analyzed in this study is subject to the following licenses/restrictions: We have a series of relevant studies in progress and need to wait until all the results are published. Requests to access these datasets should be directed to Shumei Li, lsmzssdoctor@126.com.

## Ethics Statement

The studies involving human participants were reviewed and approved by Institutional Ethics Committee of Fujian Medical University Union Hospital (Ethics approval number: 2021KY080). The patients/participants provided their written informed consent to participate in this study. Written informed consent was obtained from the individual(s) for the publication of any potentially identifiable images or data included in this article.

## Author Contributions

SL responsible for the design, manuscript drafting and overall research management. HQ statistical analysis and interpretation. ZL data collection and manuscript drafting. LF and LC guidance and content revision. YG and YZ data collection and implementation of data. LC guidance and supportive contributions. All authors contributed to the article and approved the submitted version.

## Funding

This project is supported by the funds for the National Natural Science Foundation of China (grant No 8217033).

## Conflict of Interest

The authors declare that the research was conducted in the absence of any commercial or financial relationships that could be construed as a potential conflict of interest.

## Publisher's Note

All claims expressed in this article are solely those of the authors and do not necessarily represent those of their affiliated organizations, or those of the publisher, the editors and the reviewers. Any product that may be evaluated in this article, or claim that may be made by its manufacturer, is not guaranteed or endorsed by the publisher.

## Supplementary Material

Supplementary Figure 1 The basic and postoperative dynamic changes of monocytes count (10^9^/L) **(A)** and percentage (%) **(B)** in ISR and non-ISR groups were observed. All values are presented as mean ± SD. Comparisons were conducted using the one-way ANOVA. A P value of <0.05 was considered to be statistically significant. ^#^P <0.05, ^*##*^ <0.01 vs ISR; ^*^P <0.05, ^**^P <0.01 vs non-ISR.
