# Supplementary figures and images for: The Early Predictive Value of Circulating Monocytes and Eosinophils in Coronary DES Restenosis
Source: Front Cardiovasc Med. 2022 Feb 22;9:764622. doi: 10.3389/fcvm.2022.764622 (PMC8902143; doi:10.3389/fcvm.2022.764622)

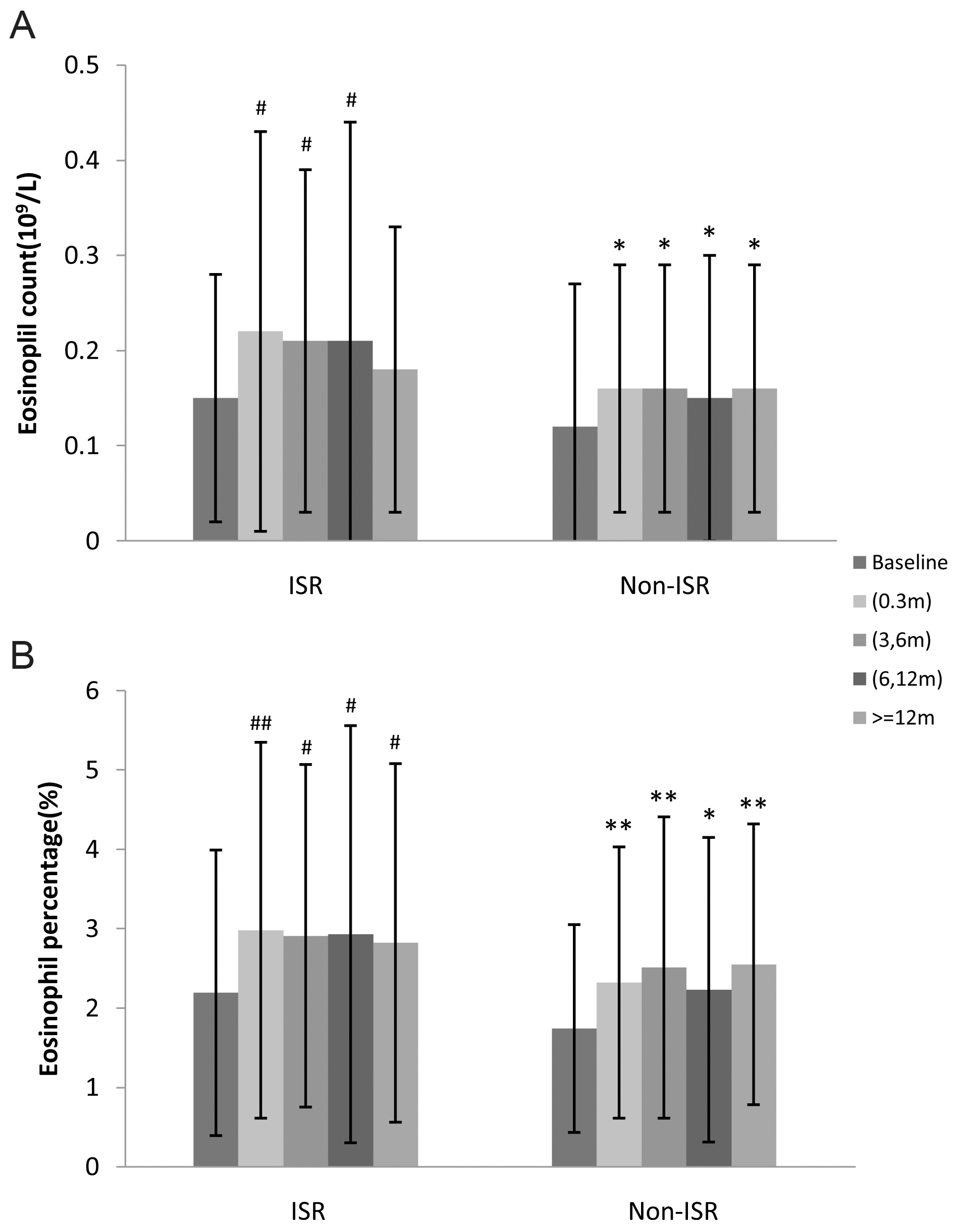

Supplement: Supplementary Figure 1 — The basic and postoperative dynamic changes of monocytes count (109/L) (A) and percentage (%) (B) in ISR and non-ISR groups were observed. All values are presented as mean ± SD. Comparisons were conducted using the one-way ANOVA. A P value of <0.05 was considered to be statistically significant. #P <0.05, ## <0.01 vs ISR; *P <0.05, **P <0.01 vs non-ISR. [file Image_1.TIF]
